# Pan-Cancer Analysis Identified *C1ORF112* as a Potential Biomarker for Multiple Tumor Types

**DOI:** 10.3389/fmolb.2021.693651

**Published:** 2021-08-19

**Authors:** Jiaxuan Chen, Haoming Mai, Haitao Chen, Bin Zhou, Jinlin Hou, De-Ke Jiang

**Affiliations:** State Key Laboratory of Organ Failure Research, Guangdong Key Laboratory of Viral Hepatitis Research, Guangdong Institute of Liver Diseases, Department of Infectious Diseases and Hepatology Unit, Nanfang Hospital, Southern Medical University, Guangzhou, China

**Keywords:** C1ORF112, prognosis, pan-cancer, biomarker, cell cycle

## Abstract

*C1ORF112* is an evolutionarily conserved gene across vertebrates. Over the last decade, studies have suggested that *C1ORF112* may play a role in tumorigenesis. Using The Cancer Genome Atlas datasets, we explored the role of *C1ORF112* across various tumor types in this study. In most tumor types, *C1ORF112* expression was increased in tumor tissues compared to corresponding non-tumor tissues. In patients with certain tumor types, higher *C1ORF112* expression was correlated with shorter overall survival, disease-free survival, and progression-free survival. Further analyses of *C1ORF112* genetic alteration data showed that *C1ORF112* amplification and mutations may have an impact on liver hepatocellular carcinoma and uterine corpus endometrial carcinoma prognosis. In cancers including lower grade glioma and adrenocortical carcinoma, *C1ORF112* expression was linked to cancer-associated fibroblast infiltration. Gene Ontology analysis showed that *C1ORF112* was co-expressed with genes involved in biological processes such as cell cycle and mitotic regulation. The protein interaction network demonstrated that *C1ORF112* physically interacted with *RAD51*, *DMC1*, and *FIGNL1*, which have well characterized functions in DNA repair and cell cycle regulation. This pan-cancer study revealed the prognostic value and oncogenic role of *C1ORF112* across multiple tumor types.

## Introduction

Cancer is the most common cause of death in the world (Sung et al., 2021). To widen the potential therapy options for malignancies, a better knowledge of carcinogenesis and tumor progression through the identification of oncogenes is crucial. Large-scale and multi-omics cancer datasets, such as The Cancer Genome Atlas (TCGA) (ICGC/TCGA Pan-Cancer Analysis of Whole Genomes Consortium, 2020; Ye et al., 2021; Lv et al., 2021; Cheng et al., 2021; Cui et al., 2020), have made pan-cancer analysis possible in the last decade.

Chromosome 1 Open Reading Frame 112 (*C1ORF112*), also known as *FLJ10706* (Howe et al., 2021), is evolutionarily conserved, especially in primates (Edogbanya et al., 2021). Mice lacking *BC055324* (the mouse ortholog for *C1ORF112*) are embryonic lethal (https://www.mousephenotype.org/data/genes/MGI:3590554), indicating that *BC055324* is required for embryonic development. According to van Dam et al., genes enriched in the BRCA–Fanconi anemia-related DNA damage response pathway, such as *BRCA1* and *FANCI*, were shown to be co-expressed with *C1ORF112* (van Dam et al., 2012). And the dysregulation of this pathway is linked to a higher risk of cancer (Nalepa and Clapp, 2018). Furthermore, *C1ORF112* knockdown inhibited cell proliferation of HeLa cells, implying *C1ORF112* may have a role in cancer (van Dam et al., 2012). Moreover, according to previous studies, *C1ORF112* was overexpressed in tumor tissues of stomach cancer and head and neck squamous cell carcinoma (Chen et al., 2020; Huang et al., 2020; Edogbanya et al., 2021). Besides, *C1ORF112* expression was found to be higher in tumor samples with mutant *TP53*, a well-known tumor suppressor, in a study of bladder cancer progression (Sanchez-Carbayo et al., 2007). To the best of our knowledge, no comprehensive analysis of *C1ORF112’*s function and clinical importance at the pan-cancer level has been done.

In this study, we systematically analyzed the expression status, prognostic value, genetic alteration, and molecular function of *C1ORF112* as well as the association with cancer-associated fibroblast infiltration in multiple tumor types.

## Materials and Methods

### Gene Expression Analysis of C1ORF112

We constructed a *C1ORF112* mRNA expression plot using the Human Protein Atlas (HPA) database (version: 20.1) (https://www.proteinatlas.org/).

The “Gene DE” module of Tumor Immune Estimation Resource version 2 (TIMER2) (http://timer.cistrome.org/) was used to investigate *C1ORF112* expression differences in tumor and non-tumor tissues in various tumor types. In this module, *C1ORF112* expression was also evaluated between distinct breast cancer molecular subgroups, between HPV-positive and HPV-negative head and neck squamous cell carcinoma (HNSC), and between primary and metastatic skin cutaneous melanoma (SKCM).

We searched *C1ORF112* in the Oncomine database (Research Edition) (https://www.oncomine.org) to generate a pooled analysis of *C1ORF112* expression.

### Survival Prognosis Analysis

Overall survival (OS) and disease-free survival (DFS) Kaplan–Meier (K-M) plots, as well as a survival significance map of *C1ORF112* in all TCGA tumor types, were generated using the Gene Expression Profiling Interactive Analysis version 2 (GEPIA2) (http://gepia2.cancer-pku.cn/) “Survival Analysis” module. In addition, UCSC Xena Browser (https://xenabrowser.net/) was used to perform progression-free survival (PFS) analysis of *C1ORF112* using TCGA Pan-Cancer datasets (version: 2018–09–13). The expression threshold was set at 50% for high *C1ORF112* expression and low *C1ORF112* expression.

### Genetic Alteration Analysis

*C1ORF112* genetic alterations were analyzed using cBioPortal (version: 3.6.20) (https://www.cbioportal.org/). Based on datasets of TCGA Pan-Cancer Atlas Studies, we calculated the frequency of *C1ORF112* gene mutation and copy number alteration in the “Cancer Types Summary” module. A mutation site plot of *C1ORF112* was created using the “Mutations” module.

To analyze the correlation between *C1ORF112* amplification status and liver hepatocellular carcinoma (LIHC) prognosis, the molecular profile was selected as copy number alterations based on “liver hepatocellular carcinoma (TCGA Pan-Cancer)” and the survival plot was generated by dividing cases based on the presence of copy number alterations (altered and unaltered groups).

To analyze the correlation between *C1ORF112* mutation status and uterine corpus endometrial carcinoma (UCEC) prognosis, the molecular profile was selected as mutations based on “uterine corpus endometrial carcinoma (TCGA Pan-Cancer)” and the survival plot was generated by dividing cases into altered and unaltered groups.

### Immune Cells Infiltration Analysis

Using the “Immune” module of Tumor Immune Estimation Resource version 2 (TIMER2) (http://timer.cistrome.org/), Extended Polydimensional Immunome Characterization (EPIC) and the Tumor Immune Dysfunction and Exclusion (TIDE) algorithms were used to investigate the correlation between *C1ORF112* expression and cancer-associated fibroblast infiltration.

### C1ORF112-Related Gene Enrichment Analysis

The STRING tool (version: 11.0b) (https://string-db.org/) was used to create a *Homo* Sapiens *C1ORF112* co-expression network using the following main parameters: 1) Active interaction sources: co-expression; 2) meaning of network edges: evidence; 3) maximum number of interactors: 50; and 4) minimum required interaction score: low confidence (0.150).

The GEPIA2 “Similar Gene Detection” module was used to extract 100 *C1ORF112*-correlated genes from the TCGA datasets that had the most similar expression pattern to *C1ORF112*. Gene Ontology pathway enrichment analysis was performed using the gene symbols of these 100 genes as input gene symbols in the “clusterProfiler” R package (version: 3.13). In addition, pairwise gene correlation analysis was performed using the GEPIA2 “Correlation Analysis” module.

### C1ORF112-Protein Interaction Analysis

The “Network” module of BioGRID (version: 4.3) (https://thebiogrid.org/) was used to create a *C1ORF112*-protein interaction network, with the layout set to “Concentric Circles.”

### Conservation Analysis of C1ORF112

The UCSC genome browser (version: 2021 update) (http://www.genome.ucsc.edu/cgi-bin/hgTracks) was used to visualize gene conversation of *C1ORF112* among vertebrates.

## Results

### Gene Expression Analysis of C1ORF112

**FIGURE 1:**
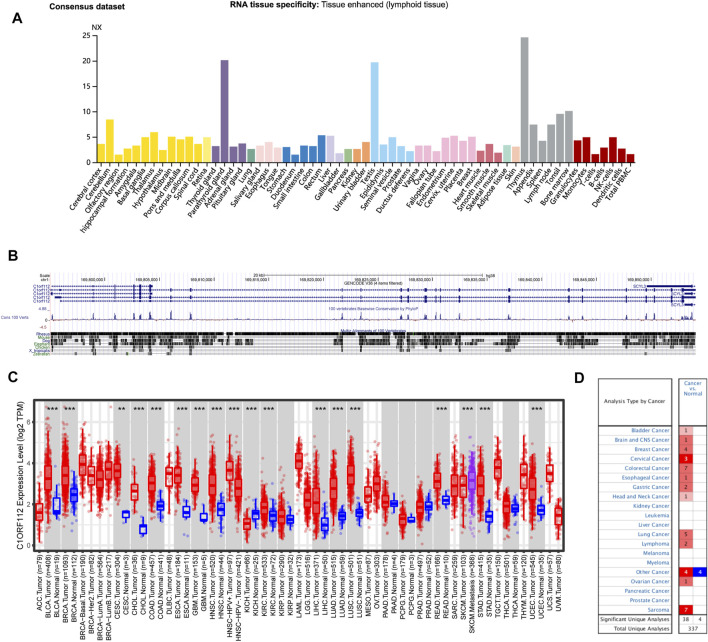
*C1ORF112* expression status in different tumors and normal tissues and *C1ORF112* gene conservation among vertebrates. **(A)** Consensus *C1ORF112* tissue expression based on datasets of HPA (Human Protein Atlas), GTEx, and FANTOM5 (function annotation of the mammalian genome). **(B)** *C1ORF112* gene conservation analysis among vertebrates was visualized using the UCSC genome browser. **(C)** The expression status of *C1ORF112* in different tumor types was visualized by TIMER2. *p < 0.05; **p < 0.01; ***p < 0.001. **(D)** Oncomine pooling analysis of C1ORF112 expression in various tumor types.

Based on datasets of the HPA, GTEx, and FANTOM5 (function annotation of the mammalian genome), we found that *C1ORF112* was highly expressed in lymphoid tissue, such as thymus and bone, and enriched in testis and the parathyroid gland (Figure 1A; Supplementary Figure S1). Moreover, based on single-cell RNA-seq, high expression of *C1ORF112* was also observed in spermatocytes and spermatogonia (Figure 1A; Supplementary Figure S1). *C1ORF112* expression has a low tissue specificity, according to these findings. We also found that *C1ORF112* is relatively conserved among vertebrates (Figure 1B).

The expression pattern of *C1ORF112* in tumor tissues was then investigated. Similar to its distribution in normal tissue, *C1ORF112* mRNA distribution also showed low tumor specificity. *C1ORF112* mRNA expression was increased in various tumor tissues when compared to corresponding normal tissue (Figure 1C). Tumor tissues of bladder urothelial carcinoma (BLCA), breast invasive carcinoma (BRCA), cholangiocarcinoma (CHOL), colon adenocarcinoma (COAD), esophageal carcinoma (ESCA), glioblastoma multiforme (GBM), HNSC, kidney renal papillary cell carcinoma (KIRP), LIHC, lung adenocarcinoma (LUAD), lung squamous cell carcinoma (LUSC), rectum adenocarcinoma (READ), stomach adenocarcinoma (STAD), UCEC (all *p* < 0.001), cervical squamous cell carcinoma, and endocervical adenocarcinoma (CESC) (*p* < 0.01) had significantly higher *C1ORF112* expression when compared to corresponding normal tissue (Figure 1C). Significantly higher *C1ORF112* expression was observed in HPV-positive head and neck squamous cell tumor tissues compared with HPV-negative tissues (*p* < 0.001) (Figure 1C). Furthermore, when compared to primary SKCM tumor tissues, *C1ORF112* expression was also significantly elevated in metastatic SKCM tissues (*p* < 0.001) (Figure 1C). Meanwhile, significantly decreased *C1ORF112* expression was observed in kidney chromophobe (KICH) tumor tissues (*p* < 0.001) (Figure 1C).

We next used the Oncomine database to validate the differential *C1ORF112* expression between tumor and normal tissues. As shown in Figure 1D, significantly elevated *C1ORF112* expression was observed in most cancer types, including bladder cancer, brain and central nervous system cancer, breast cancer, cervical cancer, colorectal cancer, esophageal cancer, gastric cancer, head and neck cancer, lung cancer, lymphoma, ovarian cancer, and sarcoma.

Together these findings suggested that C1ORF112 may promote carcinogenesis in a variety of tumor types, and its clinical significance requires further investigation.

### The Association Between C1ORF112 Expression and Prognosis of Patients With Cancer

**FIGURE 2:**
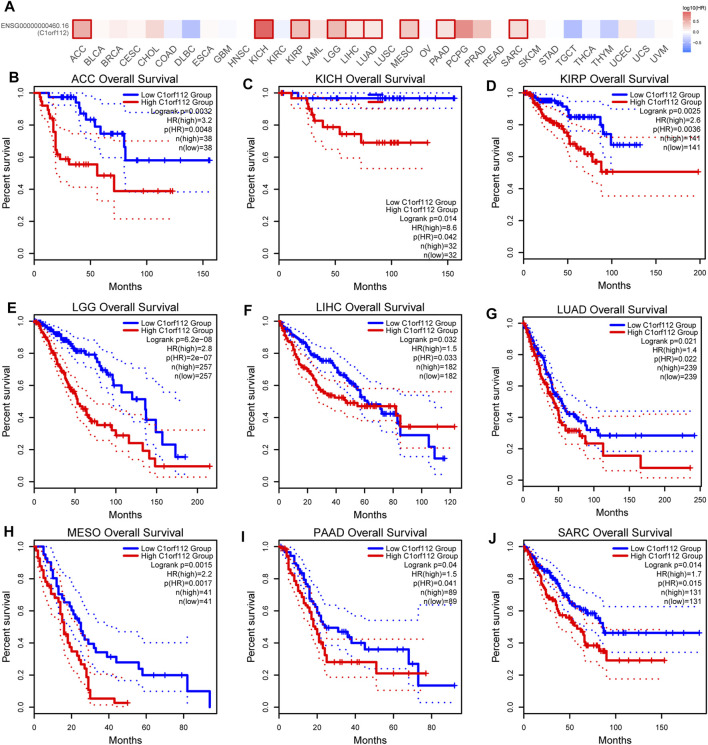
Correlation between *C1ORF112* expression and overall survival in patients with different TCGA tumor types. GEPIA2 was used to build a survival map **(A)** and conduct overall survival analyses **(B–J)**. The survival map and Kaplan–Meier plots with significant results are displayed. The 95% confidence intervals of overall survival are indicated by red and blue dotted lines for high and low *C1ORF112* groups, respectively.

**FIGURE 3:**
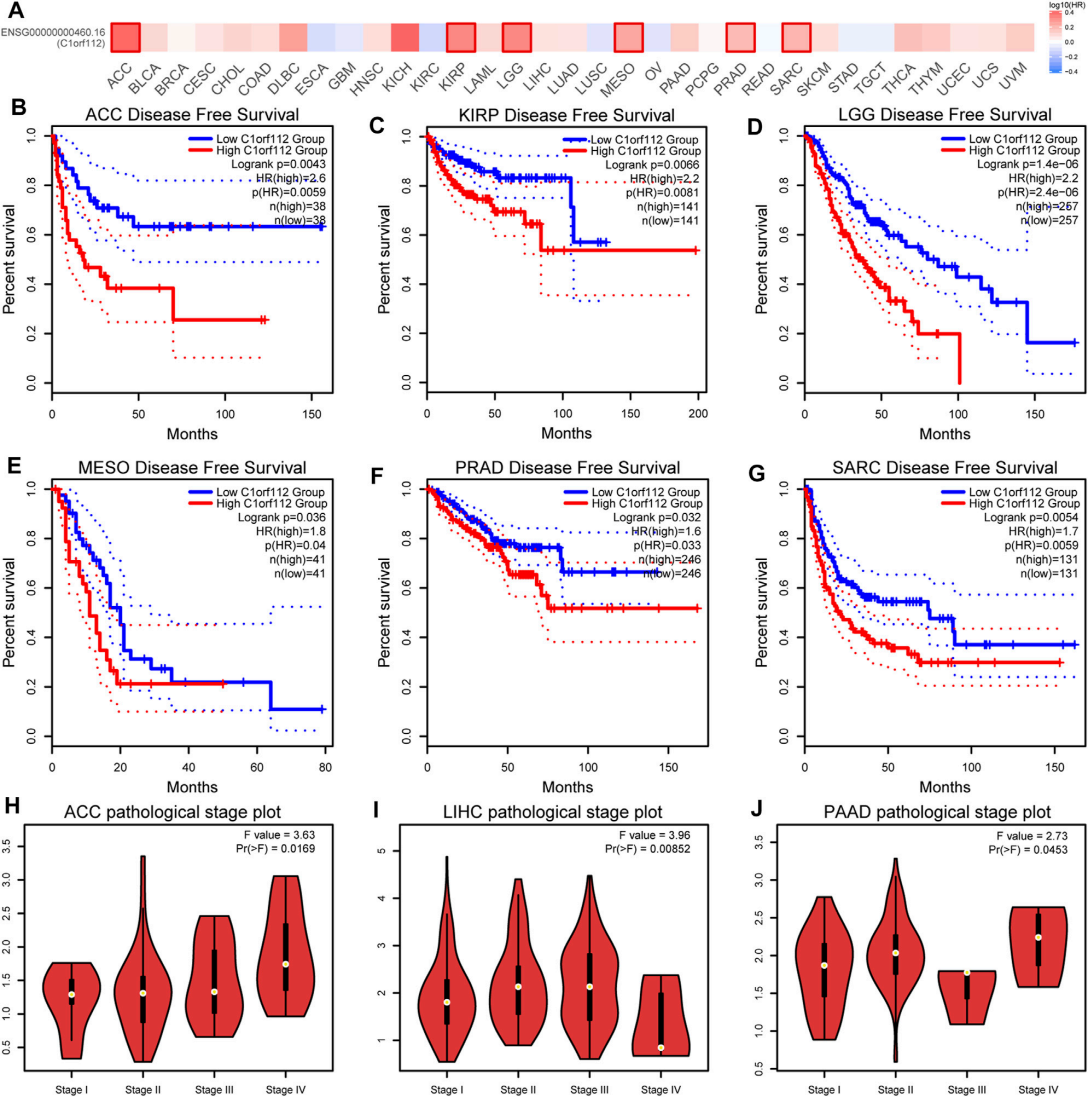
Correlation between *C1ORF112* expression and disease-free survival in patients with different TCGA tumor types. GEPIA2 was used to build a survival map **(A)** and conduct disease-free survival **(B–G)** analyses. The survival map and Kaplan–Meier plots with significant results are displayed. The 95% confidence intervals of disease-free survival are indicated by red and blue dotted lines for high and low *C1ORF112* groups, respectively. **(H–J)** Correlation between *C1ORF112* expression and pathological stages of ACC, LIHC, and PAAD from TCGA datasets. Log2 (TPM + 1) was applied for log-scale.

To explore the potential prognostic value of *C1ORF112* based on TCGA datasets, we investigated the correlation between *C1ORF112* expression and prognosis of patients with different tumors by using GEPIA2. Higher *C1ORF112* expression was associated with shorter OS in cases of adrenocortical carcinoma (ACC) (*p* = 3.2 × 10^–3^), KICH (*p* = 1.4 × 10^–2^), KIRP (*p* = 2.5 × 10^–3^), LGG (*p* = 6.2 × 10^–8^), LIHC (*p* = 3.2 × 10^–2^), LUAD (*p* = 2.1 × 10^–2^), mesothelioma (MESO) (*p* = 1.5 × 10^–3^), pancreatic adenocarcinoma (PAAD) (*p* = 4.0 × 10^–2^), and sarcoma (SARC) (*p* = 1.4 × 10^–2^) (Figure 2). Furthermore, DFS analysis showed that high *C1ORF112* expression was a marker for poor outcome for patients with ACC (*p* = 4.3 × 10^–3^), KIRP (*p* = 6.6 × 10^–3^), LGG (*p* = 1.4 × 10^–6^), MESO (*p* = 3.6 × 10^–2^), PRAD (*p* = 3.2 × 10^–2^), and SARC (*p* = 1.4 × 10^–2^) (Figures 3A–G). Using UCSC Xena Browser, a significant association was also noted between *C1ORF112* expression and PFS in several human cancers. In patients of ACC (*p* = 9.9 × 10^–4^), CHOL (*p* = 4.1 × 10^–2^), KICH (*p* = 2.6 × 10^–2^), KIRP (*p* = 3.0 × 10^–5^), LGG (*p* = 7.9 × 10^–8^), LUAD (*p* = 7.7 × 10^–3^), MESO (*p* = 9.4 × 10^–4^), PRAD (*p* = 1.5 × 10^–2^), SARC (*p* = 4.3 × 10^–2^), SKCM (*p* = 1.9 × 10^–2^), and UCEC (*p* = 5.5 × 10^–4^), higher C1ORF112 expression is associated with a worse prognosis (Supplementary Figure S2). These results indicated that increased *C1ORF112* expression was associated with poor prognosis in a variety of tumor types.

Moreover, we also investigated the correlation between *C1ORF112* expression and pathological stages of tumors by GEPIA2. The high expression of *C1ORF112* was significantly correlated with the advanced stage of ACC, LIHC, and PAAD (Figures 3H–J).

### The Genetic Alteration Landscape of C1ORF112 in Different Tumors

**FIGURE 4:**
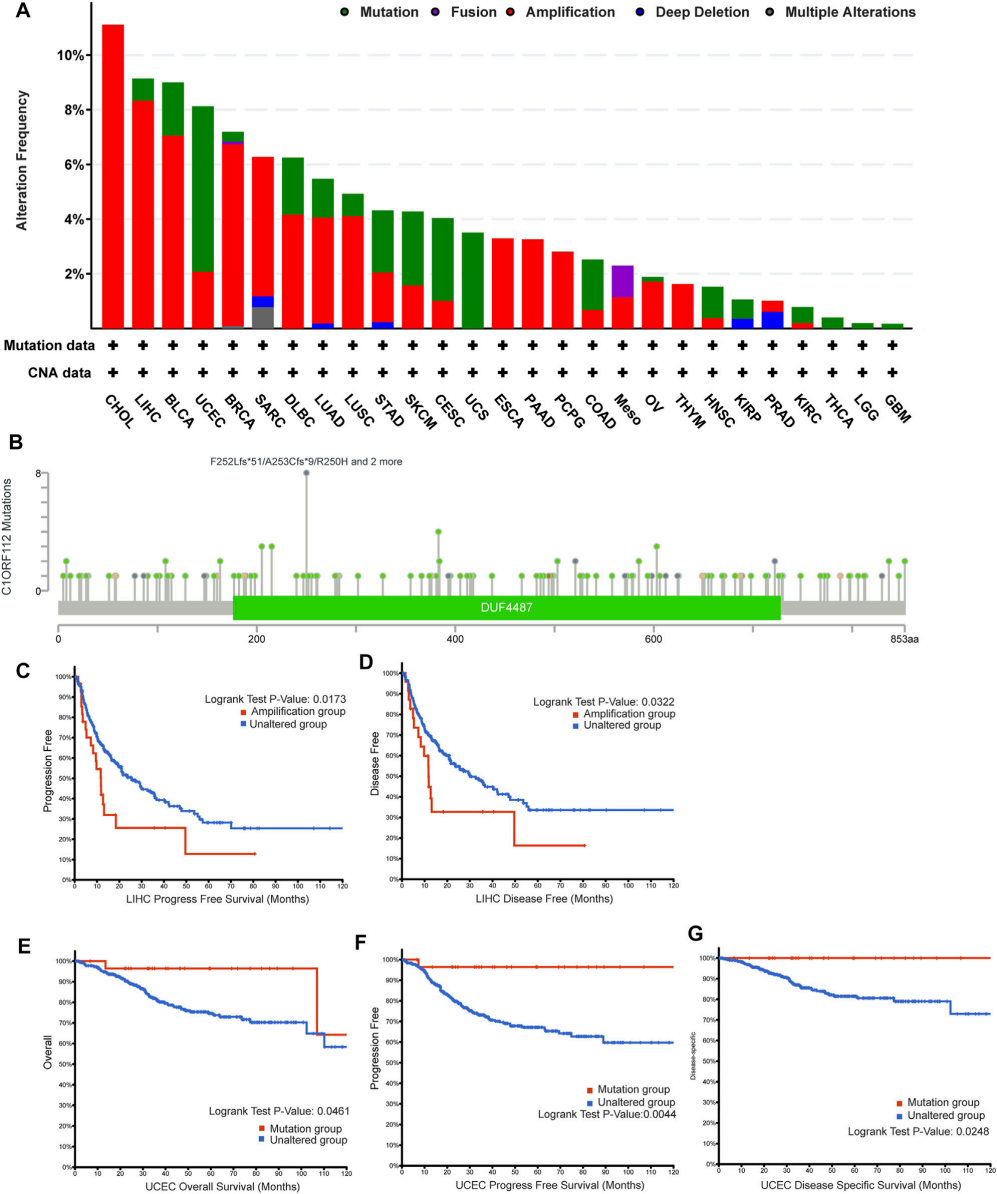
*C1ORF112* genetic alteration in various tumor types of TCGA. The alteration frequency with *C1ORF112* genetic alteration type **(A)** and *C1ORF112* mutation site **(B)** were generated by cBioPortal. The correlations between *C1ORF112* amplification status and progression-free survival and disease-free survival of LIHC **(C,D)** were analyzed by cBioPortal. The correlations between mutation status and overall survival, progression-free survival, and disease-specific survival of UCEC **(E–G)** were analyzed by cBioPortal.

The genetic alteration of *C1ORF112* in various tumor types in TCGA datasets was then investigated using cBioPortal. We found that CHOL tumor samples had the highest *C1ORF112* genetic alteration frequency (>10%). All of the genetic alterations occurring in CHOL tumor samples were copy number amplification (Figure 4A; Supplementary Table S1), which was the major type of genetic alteration in all TCGA tumor samples. In addition to CHOL cancer, more than 8% of LIHC, BRCA, and UCEC samples showed genetic alteration of *C1ORF112* (Figure 4A; Supplementary Table S1). As shown in Figure 4B, a total of 137 *C1ORF112* mutations, including 111 missense mutations, 21 truncating mutations, 4 fusion mutations, and 1 in-frame mutation, were detected in TCGA tumor samples (Supplementary Table S2). The residues 250–253 of the protein encoded by *C1ORF112* had nine mutations, making it the most frequently mutated region in the *C1ORF112* protein (Figure 4B).

Following that, we explored the link between *C1ORF112* genetic alterations and clinical outcomes of cancer patients. *C1ORF112* amplification was associated with poor prognosis in LIHC patients in terms of PFS (*p* = 1.7 × 10^–2^) and DFS (*p* = 3.2 × 10^–2^) (Figures 4C,D; Supplementary Table S3). In addition, UCEC patients with *C1ORF112* mutations (6.05%; 32 cases) showed a better prognosis in terms of OS (*p* = 4.6 × 10^–2^), PFS (*p* = 4.4 × 10^–3^), and DFS (*p* = 2.5 × 10^–2^) (Figures 4E–G; Supplementary Table S4).

### Cancer-Associated Fibroblast Infiltration Analysis

**FIGURE 5:**
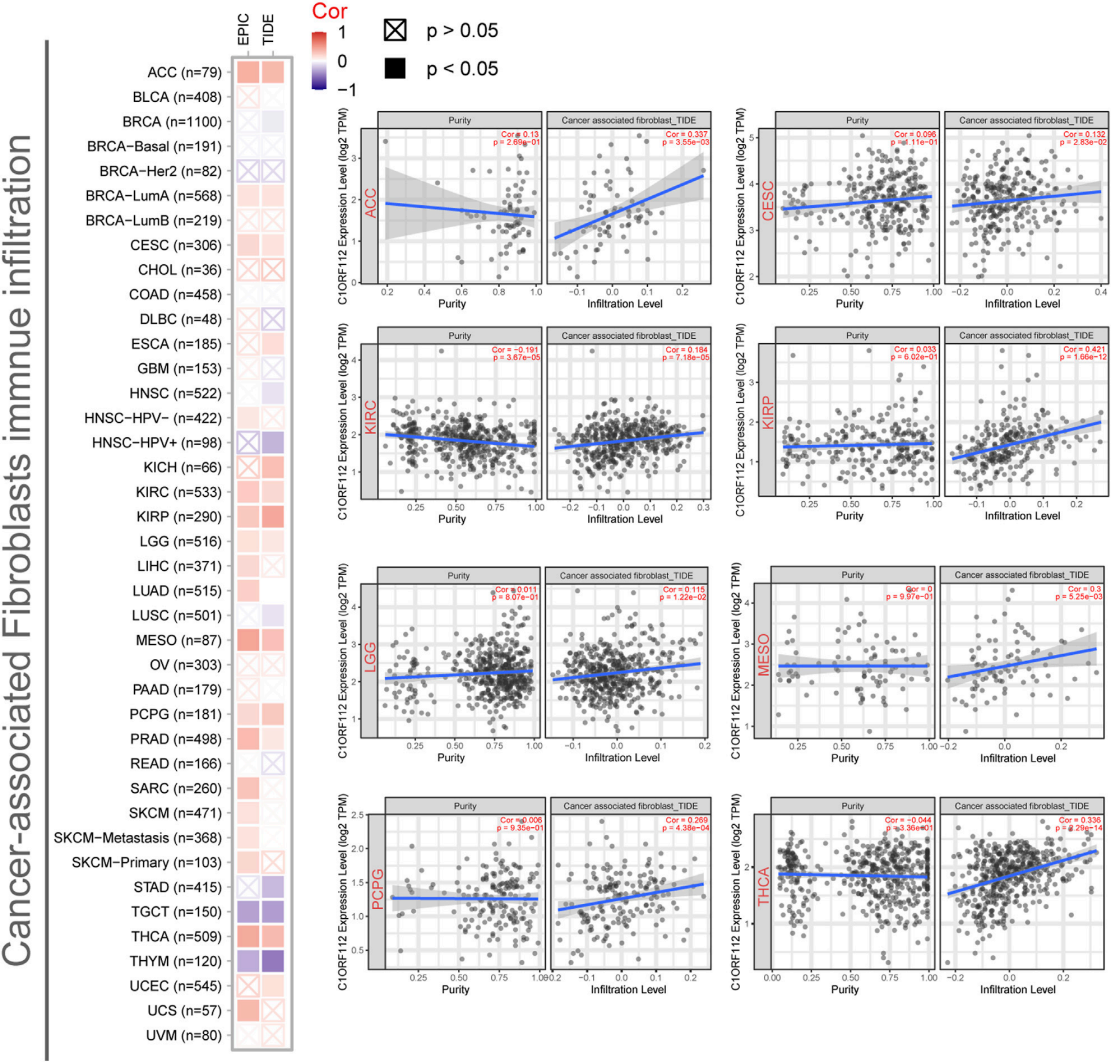
Correlation between *C1ORF112* expression and cancer-associated fibroblast immune infiltration. EPIC and TIDE algorithms were used to calculate the correlation between *C1ORF112* expression and cancer-associated fibroblast immune infiltration in all tumor types from TCGA.

Previous studies have found that cancer-associated fibroblasts in the stroma are involved in the regulation of different tumor-infiltrating immune cells (Chen and Song, 2019). We therefore employed the EPIC and TIDE algorithms to investigate the correlation between cancer-associated fibroblast infiltration and C1ORF112 expression in different malignancies. *C1ORF112* expression was positively correlated with cancer-associated fibroblast infiltration in ACC, CESC, KIRC, KIRP, LGG, MESO, and thyroid carcinoma (THCA) (Figure 5).

### C1ORF112-Related Gene Enrichment Analysis

**FIGURE 6:**
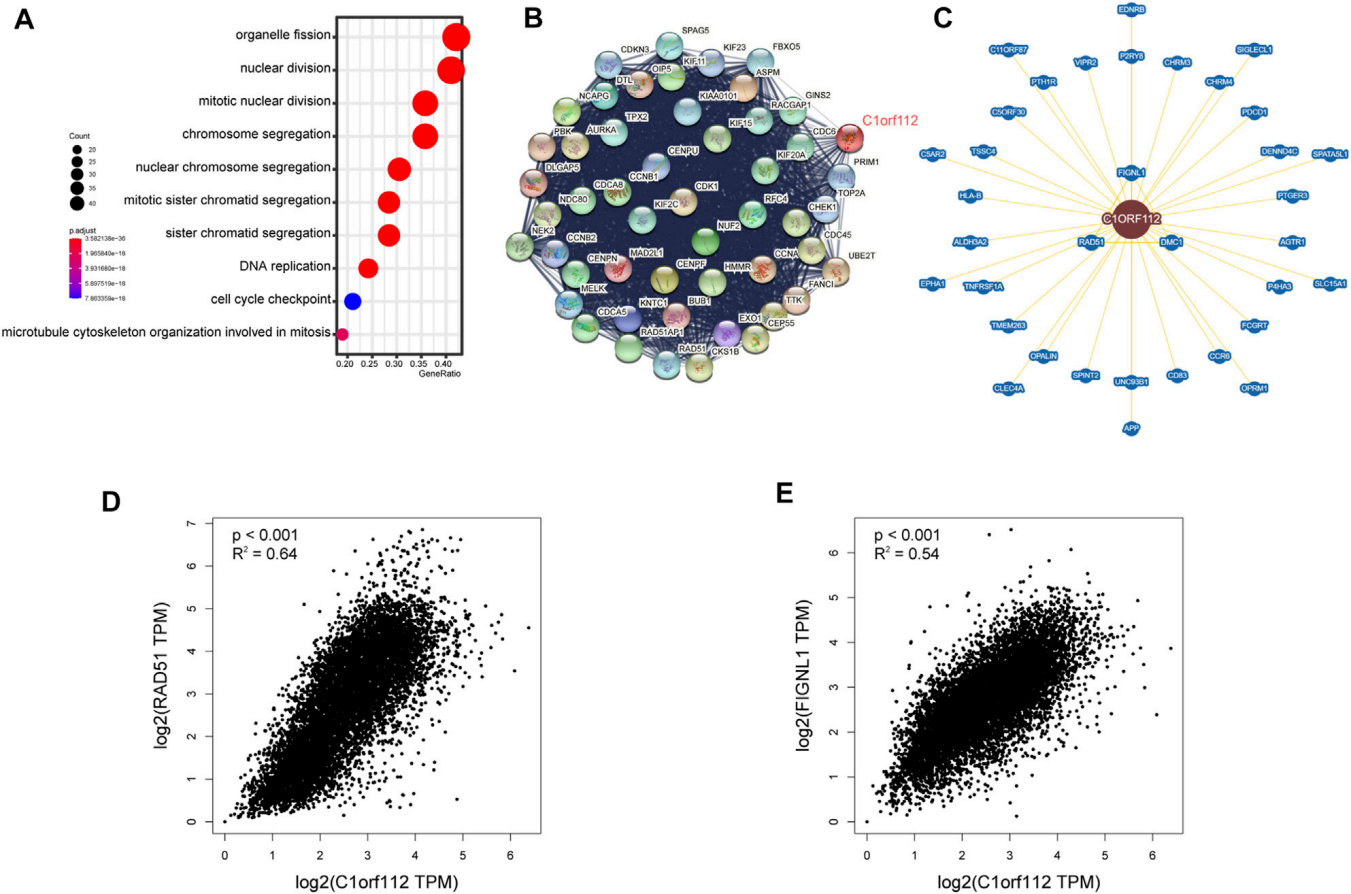
*C1ORF112*-related gene enrichment analysis. **(A)** Gene Ontology (GO) analysis of the top 100 genes co-expressed with *C1ORF112* obtained by the GEPIA2. **(B)** Co-expression network of 50 genes co-expressed with *C1ORF112* obtained by the STRING tool. **(C)** *C1ORF112*-protein interactions obtained by BioGRID. **(D,E)** Correlation analysis between *C1ORF112* and *RAD51* and *FIGNL1* conducted by GEPIA2 across all tumor samples from TCGA.

To investigate the functional mechanism of *C1ORF112* in carcinogenesis, we used GEPIA2 to extract the top 100 genes with expression patterns similar to *C1ORF112* from all tumor types in the TCGA datasets (Supplementary Table S5). Gene Ontology enrichment analysis indicated that these genes were closely linked to cell cycle or mitosis regulation (Figure 6A). Following that, 50 genes co-expressed with *C1ORF112* were obtained by the STRING tool to validate the result of Gene Ontology enrichment analysis. As shown in Figure 6B, the correlations of these 50 genes were mutually close; also, the genes were also enriched in cell cycle and mitotic regulation (Supplementary Table S6). These findings prompted us to wonder whether *C1ORF112* plays a role in these biological processes by interacting with key proteins involved in cell cycle and mitotic regulation. According to the BioGRID4.3 database, *C1ORF112* physically interacts with *RAD51*, *DMC1*, and *FIGNL1* (Figure 6C), which have well-characterized functions in the cell cycle, mitotic regulation, and tumorigenesis (Supplementary Figure S3) (Yuan and Chen, 2013; Laurini et al., 2020). Moreover, *C1ORF112* expression is strongly correlated with expression levels of RAD51 and FIGNL1 (Figures 6D,E). Based on these results, we speculate that *C1ORF112* may play a tumor-promoting role in cancers by driving the cell cycle and facilitating cell division.

## Discussion

TCGA project has profiled 33 prevalent tumor types with multi-omics data and provided an unprecedented opportunity to discover molecular aberrations at the pan-cancer level (Malta et al., 2018; Way et al., 2018; Schaub et al., 2018; Weinstein et al., 2013). Thanks to the development of bioinformatics algorithms and databases (Cerami et al., 2012; Carithers and Moore, 2013; Tang et al., 2019), numerous studies have been undertaken in recent years to identify pan-cancer molecular biomarkers and their functional roles (Ye et al., 2021; Lv et al., 2021; Hong et al., 2020). In this study, we analyzed the prognostic value and oncogenic role of *C1ORF112* in a variety of tumor types.

*C1ORF112* is located at chromosome 1q24.2 and its gene synonym is *FLJ10706*. *C1ORF112* encodes nine transcripts, five of which are protein-coding (Howe et al., 2021). The *C1ORF112* protein with the longest amino acid sequence is translated from two distinct transcripts, ENST00000286031 and ENST00000359326 (Howe et al., 2021). According to the Ensembl database, *C1ORF112* orthologues have been found in 194 species. The protein-coding sequence of *C1ORF112* has a relatively high level of conservation among vertebrates, and a previous study revealed that *C1ORF112* might have evolved from the ancestors of eukaryotes (Edogbanya et al., 2021). Mice lacking *BC055324* (the mouse ortholog of *C1ORF112*) are embryonic fatal, and heterozygous mice have lower bone mineral density, circulating glucose level, and cardiac output (https://www.mousephenotype.org/data/genes/MGI:3590554). In the last decade, *C1ORF112* has drawn attention for its potential role in tumorigenesis. Van Dam et al. found that *C1ORF112* knockdown in HeLa cancer cells significantly lowered cell growth rate (van Dam et al., 2012). A recent study revealed that *C1ORF112* was co-expressed with stem cell-related genes, and these genes had elevated expression in gastric cancer tissues (Huang et al., 2020). Another study identified *C1ORF112* as one of the genes in a nine-gene risk model for predicting prognosis in patients with gastric cancer (Chen et al., 2020). However, the significance of *C1ORF112* in various tumor types has not been comprehensively explored. Therefore, we systemically characterized *C1ORF112* in 33 TCGA tumor types by analyzing features such as gene expression, genetic alteration, and immune infiltration.

In the present study, we found that *C1ORF112* is widely expressed in a variety of tissues and *C1ORF112* expression is upregulated in the majority of tumors. We further explored the relationship between *C1ORF112* overexpression and clinical parameters or prognosis. Survival analysis revealed that *C1ORF112* overexpression was associated with poor OS, DFS, and PFS. High *C1ORF112* expression was associated with poor prognosis in different tumor types involving ACC, CHOL, KIHC, KIRP, LGG, LUAD, MESO, PAAD, and SARC. Furthermore, upregulated *C1ORF112* expression is also significantly associated with the advanced cancer stage suggesting malignant progression. Growing evidence indicates that genomic mutations involved tumor progression and chemotherapy response (Yang et al., 2011; Cha et al., 2021). For example, Yang et al. reported that *BRCA1* and *BRCA2* mutations are significantly associated with patient survival*,* which may be a result of distinct response to platinum-based treatment (Yang et al., 2011). A large-scale study identified that mutations in four genes (ESR1, CDH1, RICTOR, and TP53) tended to occur in specific metastatic sites, which could be biomarkers or therapeutic targets of metastatic breast cancer patients (Cha et al., 2021). In this study, we revealed that mutations of *C1ORF112* were most common in CHOL (>10%), followed by LIHC, BRCA, and UCEC. To analyze whether *C1ORF112* genetic alterations have an impact on clinical outcomes of cancer patients, we discovered that *C1ORF112* amplification could be a risk factor for patients with liver cancer, while *C1ORF112* mutation may be protective in UCEC patients. Collectively, these findings indicate that *C1ORF112* acts as an oncogene in the progression of a variety of cancers and is a promising predictor for practical application in cancer prognosis.

Immune cells extensively intertwine with cancer cells and exert an essential effect on cancer migration and metastasis in various tumor types (Angelova et al., 2015). Recent studies have also reported that tumor immune microenvironment was associated with the expression level of various genes (Ju et al., 2020a; Ju et al, 2020b). In this study, we found *C1ORF112* expression was positively correlated with CAFs infiltration in several tumor types. CAFs are prominent components of stromal cells and have been reported to be associated with worse prognosis, chemotherapy resistance, and disease recurrence in various cancers (Calon et al., 2015; Ryner et al., 2015; Liu et al., 2016; Fiori et al., 2019). Taken together, our work elucidates the underlying effect of *C1ORF112* in tumor immunity and its prognostic values for multiple cancers.

Using STRING and GEPIA2, we identified a number of genes that were co-expressed with *C1ORF112* across different tumors and other tissues. Gene enrichment analysis revealed that these genes were strongly correlated with cell cycle or mitosis regulation, which was consistent with previous studies (van Dam et al., 2012; Edogbanya et al., 2021). Moreover, our results showed that *C1ORF112* physically interacts with *RAD51*, *FIGNL1*, and *DMC1*. And the expressions of *RAD51* and *FIGNL*1 were strongly correlated with *C1ORF112* expression. *RAD51*, *FIGNL1*, and *DMC1* are well-characterized genes that encode proteins involved in DNA repair and cell cycle regulation (Yuan and Chen, 2013; Laurini et al., 2020). These findings validated the results of our gene enrichment analysis and paved the way for further exploration of *C1ORF112* molecular function.

This study had several limitations as well. First, the sample sizes for some uncommon tumor types were relatively small, which may cause batch effects or inaccurate results. Second, this study only provides preliminary findings linking *C1ORF112* to cancer progression in various tumors, and more experimental work is needed to determine the precise molecular function of *C1ORF112* in tumorigenesis.

In conclusion, *C1ORF112* is widely overexpressed in diverse cancers and its expression and genetic alteration are statistically associated with clinical outcomes in patients with certain tumors. Furthermore, immune infiltration analysis and *C1ORF112*-related gene enrichment analysis offer potential mechanisms by which *C1ORF112* may regulate tumor immunity, cell cycle, or DNA repair in cancers. Hence, further experimental and clinical studies are warranted to investigate *C1ORF112’s* practical application in cancer therapy and prognosis prediction.

## Data Availability

The datasets presented in this study can be found in online repositories. The names of the repository/repositories and accession number(s) can be found in the article/Supplementary Material.
