# Supplementary material for: Pan-Cancer Analysis Identified C1ORF112 as a Potential Biomarker for Multiple Tumor Types
Source: Front Mol Biosci. 2021 Aug 19;8:693651. doi: 10.3389/fmolb.2021.693651 (PMC8416665; doi:10.3389/fmolb.2021.693651)
Supplement: Supplementary file 1 [file DataSheet1.ZIP › Supplementary Material_20210713/Supplementary Figure.docx]

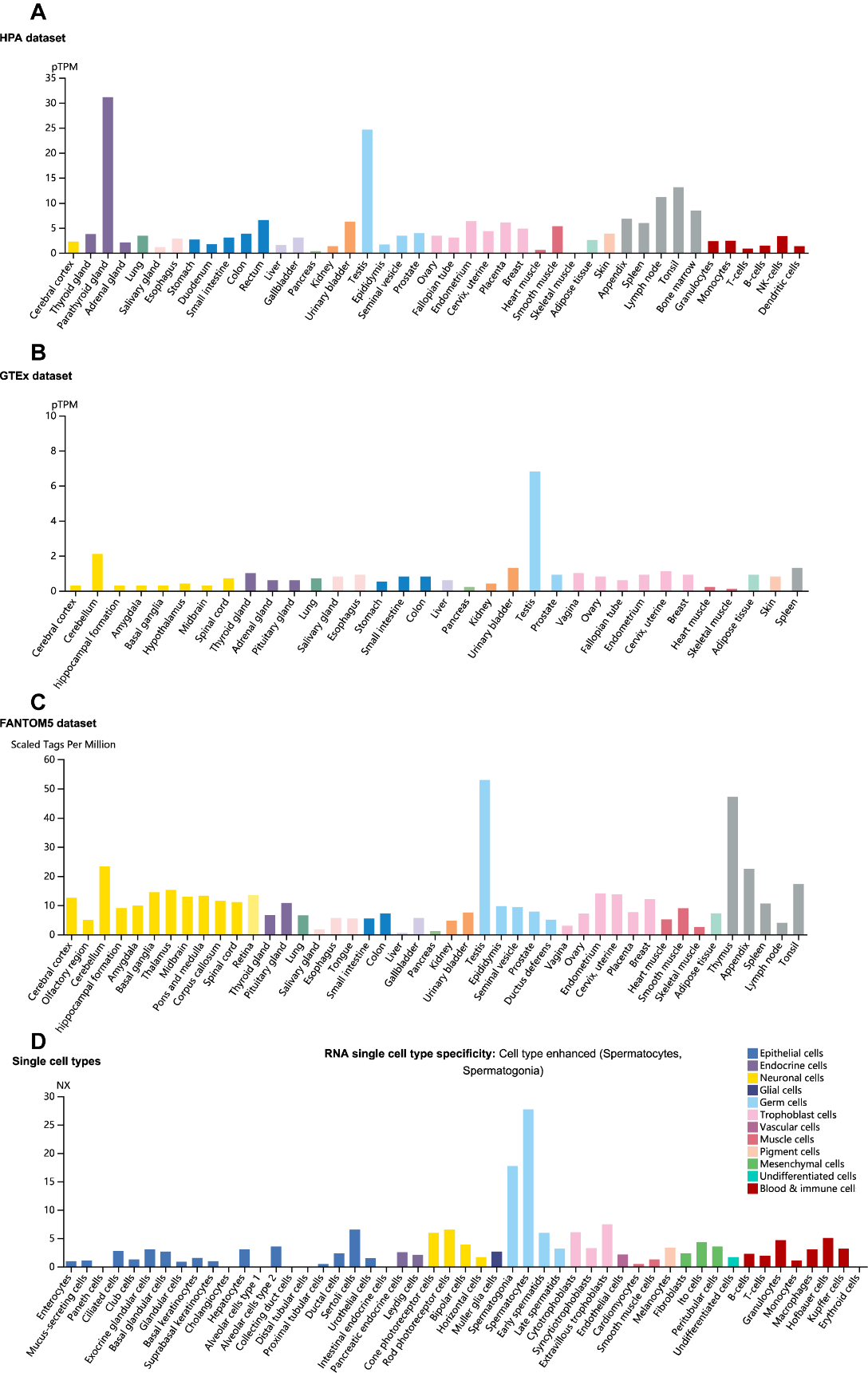


Supplementary Figure 1. C1ORF112 expression status in different normal tissues. (a-c) C1ORF112 tissue expression based on datasets of the HPA (Human protein atlas), GTEx, and FANTOM5 (Function annotation of the mammalian genome 5). (d) C1ORF112 expression in various cell types.


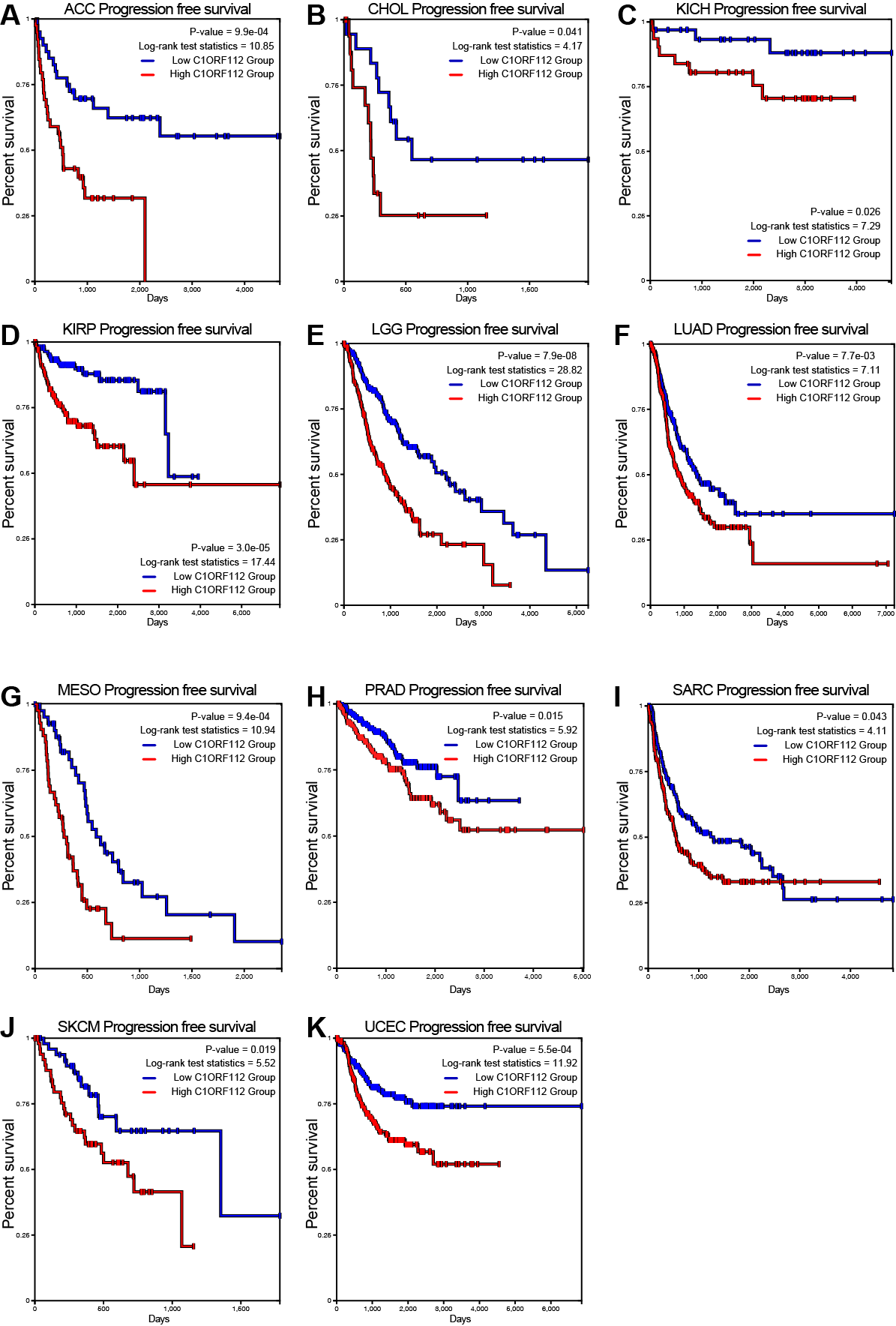
Supplementary Figure 2. Correlation between C1ORF112 expression and progression-free survival in patients with different TCGA tumor types. UCSC Xena browser was used to build conduct progression-free survival analyses (a-k). Kaplan-Meier plots with significant results are displayed.


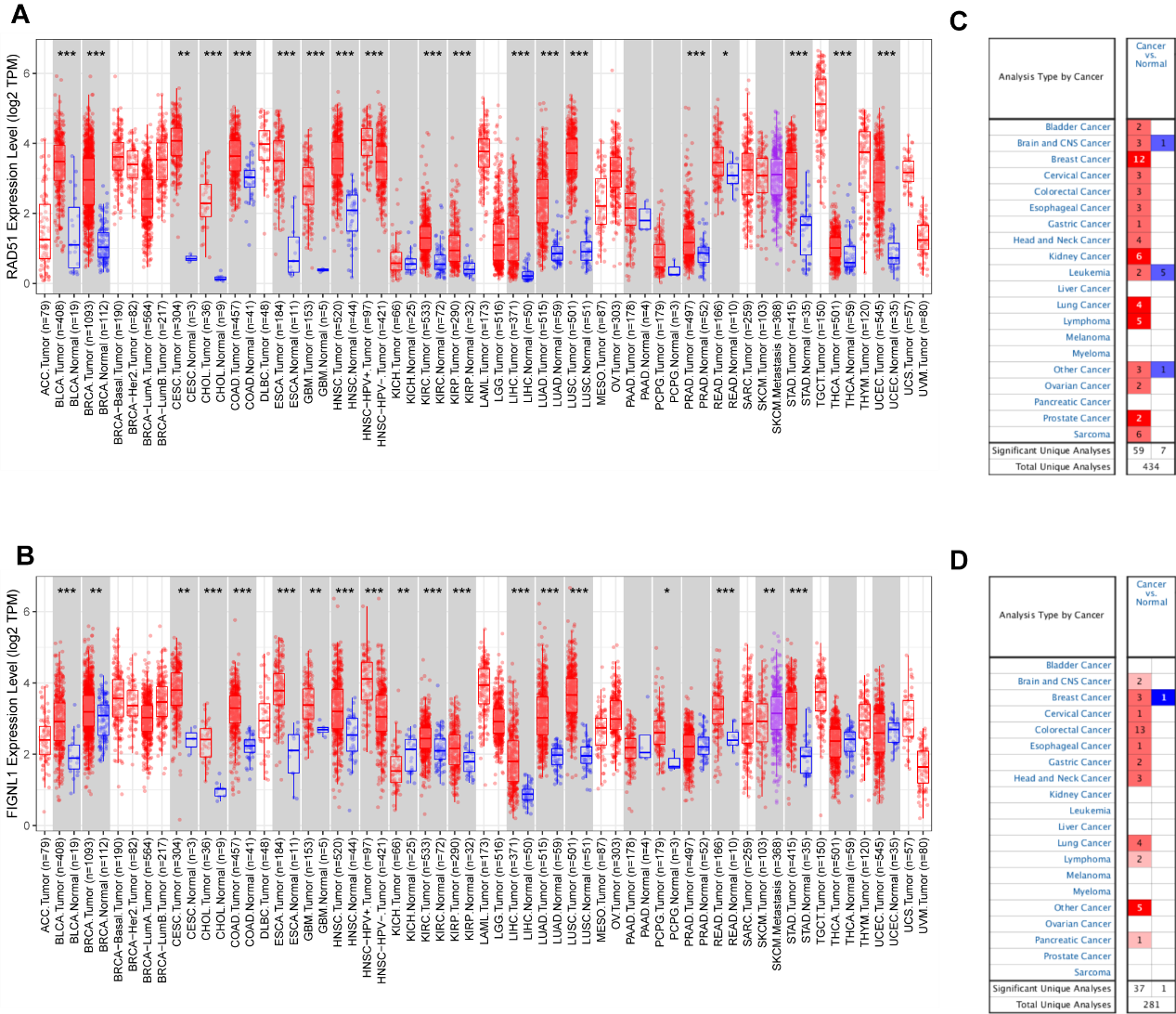


Supplementary Figure 3. RAD51 and FILGN1 expression status in different normal tissues.

(a-b) The expression status of RAD51 and FILGN1 in different tumor types was visualized by TIMER2. * P < .05; ** P < .01; *** P < .001. (c-d) Oncomine pooling analysis of RAD51 and FILGN1 expression in various tumor types.
